# Bex-Nikaidoh concept for repair of d-transposition of the great arteries with ventricular septal defect and pulmonary stenosis or pulmonary atresia

**DOI:** 10.1186/1749-8090-8-S1-O252

**Published:** 2013-09-11

**Authors:** A Chernogrivov, V Bazylev, I Chernogrivov, J Kalinicheva, SH Suleymanov, T Rybakova, O Talysheva, T Nevvazhay

**Affiliations:** 1 Federal Center for Cardio-vascular Surgery, Penza, Russia

## Background

This study analyzes the results of Bex-Nikaidoh procedure in midterm follow-up for transposition of the great arteries with ventricular septal defect and pulmonary atresia. We also used the concept of Bex-Nikaidoh for former combination of defects with pulmonary atresia.

## Methods

Between 2008 and 2013 seven patients underwent modified Bex-Nikaidoh at median (M) age of 7.5 months (CI 25-75% [4.2; 26.4]). The conal septum was divided in 4 (CI 25-75% [44.4; 68.9]) patients including 1 patient with the surgical creation of outflow tract of the left ventricle due to pulmonary atresia. Modified technique of aortic root translocation included replantation of coronary arteries and placement into the ortotopic position of “oversized” pulmonary ventricle–pulmonary artery conduit. The evaluation of conduit performance was based on transthoracic and transesophageal echocardiogram and computed tomography.

## Results

There was one hospital death (sepsis) and no late deaths or reoperations at midterm follow-up M=21 months (CI 25-75% [12; 44]) (maximum 56 months). All patients had markedly “oversized” conduits with the median Z-score 10.8 at implantation. Later there was gradual decreasing of homograft size mismatch for body surface area to median Z-score 8.7 (p=0.02) at last examination. Pulmonary conduit peak gradient did not progress in all patients over time (M=3.5 vs 4 mm Hg, p=0.85). There was no aortic root dysfunction – median peak gradient was 4 mm Hg at discharge and 6 at follow-up (p=0.35).

## Conclusions

We used the concept of Bex-Nikaidoh operation additionally for transposition of the great arteries with ventricular septal defect and pulmonary atresia. It makes it possible to implant the homograft of larger diameter than the other known techniques in all cases simultaneously resulting in an excellent hemodynamic performance of left ventricle outflow tract.

